# A cluster randomized controlled trial comparing the effectiveness of an individual planning intervention with collaborative planning in adolescent friendship dyads to enhance physical activity (TWOgether)

**DOI:** 10.1186/s12889-018-5818-6

**Published:** 2018-07-24

**Authors:** Theda Radtke, Aleksandra Luszczynska, Konstantin Schenkel, Stuart Biddle, Urte Scholz

**Affiliations:** 1 Department of Psychology, Applied Social and Health Psychology, University of Zurich, Binzmuehlestrasse 14/14, 8050 Zurich, Switzerland; 2 Institute for Resilient Regions, University of Southern Queensland, Education City, 37 Sinnathamby Boulevard, Springfield Central, QLD 4300 Australia; 3 SWPS University of Social Sciences and Humanities, 30b Ostrowskiego Street, PL-53-238 Wroclaw, Poland; 4 Trauma, Health, & Hazards Center, University of Colorado at Colorado Springs, 1420 Austin Bluffs Pkwy, Colorado Springs, CO 80918 USA

**Keywords:** Action planning, Coping planning, Collaborative planning, Adolescents, Physical activity, Individual self-regulation strategies, Social exchange processes, Dyads, Ecological momentary assessment

## Abstract

**Background:**

Most adolescents do not meet the recommendations for physical activity (PA) of at least 1 h per day. Individual planning (IP) interventions, including forming plans for when, where and how (action planning) to engage in a behavior, as well as the planning for how to deal with arising barriers (coping planning), are effective to enhance PA in adults. Collaborative planning (CP) is conjoint planning of two individuals regarding a behavior which is performed together. It is assumed that CP stimulates social exchange processes between the planning partners. However, it remains unclear whether planning interventions of PA in adolescents are successful and which planning intervention is more effective. Thus, this cluster randomized controlled trial (RCT) examines changes in daily moderate-to-vigorous PA in adolescents’ friendship dyads resulting from planning. Individual self-regulating mechanism and social exchange processes are proposed as mediating mechanisms of the effects of planning for health behavior change.

**Methods:**

A single-blind four-arm parallel-group cluster-RCT is used. The sample consists of 400 friendship dyads between 14 and 18 years of age. As the recruitment takes place in schools, a cluster randomization of the schools is used to enroll dyads to (a) an IP intervention, (b) a CP intervention or (c) one of the two no-planning control conditions. Devise-measured and self-reported PA as the primary outcomes, self-regulatory strategies, and social exchange processes as secondary outcomes are assessed at three or four time points. After baseline measurement, the baseline ecological momentary assessment of the main variables takes place for 8 days followed by the intervention and a 7-days diary phase. Follow-ups are 1 month and 6 months later. Subsequent to the six-month follow-up, another 7-days diary phase takes place.

**Discussion:**

This is the first study examining IP in comparison to CP in adolescents applying a single-blind cluster RCT. Consequently, the study allows for understanding the efficacy of individual and collaborative planning and the underlying mechanisms in adolescent dyads.

**Trial registration:**

This RCT was funded by the Swiss National Science Foundation (100019_169781/1) and was registered on 18/06/2018 at ClinicalTrials.gov: NCT03575559.

**Electronic supplementary material:**

The online version of this article (10.1186/s12889-018-5818-6) contains supplementary material, which is available to authorized users.

## Background

### Physical activity

Non-communicable diseases such as coronary heart disease, type 2 diabetes, or certain types of cancer are already major global challenges, and will become even more important to national health systems, including the Swiss health system [1]. As physical inactivity has been identified as one of the main risk factors for the main non-communicable diseases as well as the fourth leading risk factor for global mortality [2], the promotion of physical activity (PA) in adolescents is an important goal of the National Programme of Nutrition and Physical Activity of the Swiss Federal Office of Public Health (SFOPH) [3].

For children and adolescents in Switzerland at least 1 h of moderate-to-vigorous physical activity (MVPA) per day is recommended [4]. However, studies such as the Health Behavior in School Aged Children (HBSC) have shown that only a very low percentage of adolescents (12–19% of the boys; 6–11% of the girls) living in Switzerland is moderate-to-vigorous physically active for at least 1 h per day at 7 days per week. Boys turn out to be significantly more active than girls and, consistent with other studies, the activity levels of young individuals decrease with their age e.g., [1, 5]. These data, therefore, highlight the importance of physical activity interventions for this population.

### Planning intervention techniques

One of promising intervention strategies is planning [6–8]. Planning is a very simple strategy with impressive effects, as indicated by medium to large effect sizes on behavior observed across various populations and behaviors [6, 9–12]. The most important planning intervention techniques are *implementation intentions* [9] and *action planning*^1^ [10]. According to a recent consensus paper, planning is defined as “one means to attain goals. Implementation intentions are a form of planning that specify a critical condition linked to goal-directed response.” [13]. When planning individually, a person is linking a situational cue (when/where) to an intended behavioral response (how) by mental simulation of anticipated situations [9]. Thus, it is aimed to perform a link between a specific cue and an intended action to translate goal intentions into behavior. An example is “If situation *Y* is encountered, then I will initiate the goal-directed behavior *X*!” In addition, planning is often complemented by coping planning (anticipation of barriers and the formation of plans how to overcome them [14]). Regarding PA, two meta-analyses including more than 40 studies supported the effect of individual planning to increase PA [15, 16].

### Planning in children and adolescents

Recent studies indicate great promise for the effectiveness of planning in adolescents (e.g., [17–23]). For example, Armitage and Sprigg [18] showed that planning significantly increased PA compared to a control condition in eight-year-old children with low socioeconomic status. Likewise, in another study on PA in 13-to-17 olds, planning was found to be the most important predictor of adolescents’ PA [21]. In a study by Araújo-Soares et al. [24] adolescents in the planning intervention reported 57 min more physical activity per week at the 9-month follow-up compared to the control group. Moreover, as a result of developing concrete plans about how to deal with barriers for regular physical activity, adolescents in the intervention group showed significant improvement in coping planning than controls. Since the plans for PA were created together with all the class mates, the authors assumed that conjoint planning explains the effect of the intervention through a change of the social norm within the school classes. However, some experimental studies showed that individual planning may have very small effects on adolescents’ PA [25]. Hence it is necessary to explore if other types of planning may have stronger effects.

### Collaborative planning

To date, most research on planning focuses on individual planning. Typically, participants form individual plans on their own or by using planning sheets [26]. However, one promising addition to individual planning is planning in conjunction with others, namely collaborative and dyadic planning^2^ [27–31]. Collaborative planning is defined as conjoint planning of at least two individuals when, where and how to perform a behavior together [29]. An example would be “If we go shopping, we will take the bicycles to get there”. In contrast, dyadic planning implies creating plans together with a partner, but executing the behavior individually [28].

So far, research is rare regarding conjoint planning even though the findings are promising for health behavior change. Moreover, an expert group reported high consensus on the need for further studies in this field [13]. Prestwich et al. [30, 31] showed the effectiveness of collaborative planning in contrast to individual planning to enhance PA and to reduce body weight. As outlined by the studies investigating conjoint planning, the effects of conjoint planning were mediated by perceived social influences, such as provided and received partner support or social control [27, 28, 32]. Furthermore, Prestwich et al. [29] assumed that collaborative planning is mediated by reduced forgetfulness on behavior change. Therefore, the enhanced effects of collaborative planning (as compared to individual planning) may be expected as the effects of planning interventions are limited due to memory decay and cognitive interference over time [33]. Despite these first results, the evidence regarding the mechanism of the effects of conjoint planning is limited, especially regarding adolescents. Preliminary evidence indicates that collaborative planning may have stronger effects on PA in adolescents compared to individual planning [17]. In addition, a review showed that the collaborative PA engagement of an adolescent with his/her best friend is associated with higher PA intensity and an increased PA motivation [32]. This supports the argument that the peer group may be an appropriate context in which PA interventions of collaborative planning can be applied.

### Importance of social factors on health behavior

The influence of social networks on PA in adolescence has been widely investigated e.g., [34–36]. Nevertheless, studies on social networks do not provide knowledge about the underlying mechanisms responsible for changes in PA. Thus, studies investigating social exchange processes as potential mechanisms are needed.

Social exchange processes are interactions between individuals influencing an individual’s behavior, emotions, and cognitions. A central component of social exchange processes is social support [37]. In general, social support can be defined as the assistance of significant others in times of need. Furthermore, several types of social support have been investigated, such as instrumental (e.g., driving the adolescent to the sport club), informational (e.g., give advice) and emotional support (e.g., give consolation) [37, 38]. Moreover, perceived (anticipated help in time of need) and received support (help provided within a given time period) may be distinguished. Perceived social support is regarded more as a personality trait whereas received social support is based on actual support transactions in the past [38].

Perceived peer support for PA in adolescents has been extensively studied, as the influence of peers and friends becomes increasingly important due to the increased autonomy in adolescence. Results showed that peer support was related to increased PA as well as increased self-efficacy [34]. However, studies on peer support in adolescents have several limitations. First, most studies are cross-sectional [32, 34]. Second, when peer social support is measured, commonly a total score of perceived social support is used, thereby limiting the ability to determine the unique influences of different types of social support on PA [39]. Third, even though some studies showed that fostering social support (e.g., via buddy systems) is effective to enhance PA (albeit mainly in adults), the effects were rather small, and temporary or inconsistent e.g., [40]. Furthermore, there is a lack of studies examining whether peer social support is a mediating mechanism between the effect of interventions aiming to enhance social support (e.g., via buddy systems) and the PA in adolescents. Thus, several open issues remain which are addressed in this study.

To date, the experimental investigation of the mechanisms and moderators of the effects of planning on PA in young people is limited e.g., [20, 21, 23] and needs further investigation. Moreover, longer-term effects with follow-ups of more than one month e.g., [19] are understudied, assessment of behavior using wearable technology is rare, and the vast majority of planning studies in adolescence have focused on individual planning only. Therefore, the consideration of all these aspects is important to identify the most promising planning intervention [7, 13].

## Aim

The aims of the present study are threefold. First, the effectiveness of planning moderate-to vigorous physical activity is compared against two control groups on devise-based assessment of MVPA. Beside this main aim, it is also aimed at specifically comparing the effectiveness of collaborative and individual planning for PA in adolescents. Third, this study examines the assumed underlying mechanisms of these planning interventions, social exchange as well as individual self-regulatory processes (e.g., self-efficacy). The study design allows examining micro- and macro-time changes in outcomes as well as potential mediating mechanisms using a daily diary assessment together with devise-based assessment of PA after the intervention as well as in the long-run. For the hypotheses related to these aims, please see the trial registration: ClinicalTrials.gov: NCT03575559.

## Methods/Design

**Fig. 1:**
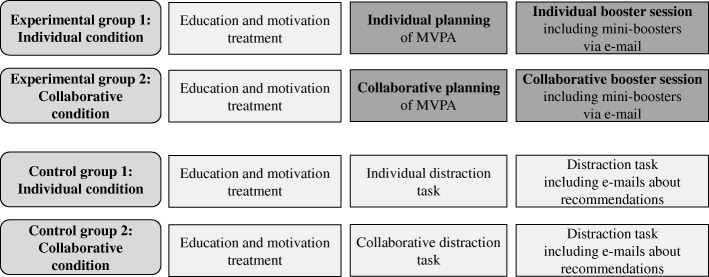
Experimental design. Note. MVPA = Moderate-to-vigorous physical activity

The study is planned as a single-blind four-arm cluster-randomized controlled planning intervention with a longitudinal design with micro- and macro-time assessments (cf. Fig. 1).

### Sample and recruitment strategy

Adolescents and one of their closest friends (friendship dyads) between 14 and 18 years old is recruited. Inclusion criteria for participation require a moderate to strong intention for PA as well as an actual MVPA of the adolescents which is below the recommended amount of 1 h or more per day [4]. Furthermore, the friendship dyads have to be of the same gender composition. In addition, friendship dyads which are in romantic relationship are excluded [41]. Both adolescents also need to agree on participating in the study. In case of adolescents under 16 years of age, their parents or legal guardians provide written informed consent about the participation of their child. In order to be able to complete the daily diaries, both adolescents in the dyad need to have internet access at home or mobile internet via their smartphone. Exclusion criteria include conditions which prohibit or restrict participants from being physically active or doing exercise (e.g., asthma; according to the Physical Activity Readiness Questionnaire [42]), an age and sex adjusted BMI below 17 [43], pregnancy, a romantic relationship with the participating friend, insufficient knowledge of the German language, and participation in other research studies targeting physical activity.

Recruitment take place in public schools in the German speaking regions of Switzerland. The efficacy of this recruitment strategy was indicated by a pilot study that was conducted in January 2016. Recruitment is organized via flyer, postings in schools, and social media (e.g., Facebook groups of the schools). In addition, trained experimenters present the study during school lessons. The intervention is advertised as a study about enhancing physical activity together with a close friend using the catchphrase “TWOgether – From Sport Zero to Sport Hero”. Beside the flyers and posters raising awareness about the study, a website (https://twogetherstudy.jimdo.com/) can also be consulted for further information.

### Randomization

As the recruitment takes place in public schools a cluster randomization of participants to the four study conditions is necessary to avoid communication between the students about the conditions. In addition, a school-balanced randomization is used to minimise imbalance in the group assignment regarding the school type (i.e., “Gymnasium” which is the highest school level (Level A), “Sekundarschule” which is equivalent to secondary school (Level B or C); and “Berufsschule” or vocational school where young people receive a practical and vocational training related to their job). For this reason, blocking as means of restricted randomization is used. Within a block the three participating school types are assigned to one of the four groups.

A computerized random-number generator is used for the generation of the cluster and block randomized allocation of the participants (randomizer.org). The allocation sequence is generated by a researcher who is not involved in the data collection process. In addition, the allocation to the study conditions is securely stored. A research assistant prepares sealed, numbered envelopes to ensure that group assignment remains unknown to the experimenters. Only 2 days before the intervention session (T2) the experimenters are allowed to open an envelope indicating the assignment of the dyad to the study condition to prepare the study materials in advance.

### Procedure

#### Pre-screening and baseline (T1)

Adolescents interested in the study first fill in an online pre-screening questionnaire with the inclusion and exclusion criteria. If all inclusion criteria are met, the friendship dyad is contacted by a member of the study staff (e.g., via phone/e-mail) to make an appointment for the first measurement session. In case of adolescents under 16 years of age, a written signed parental informed consent has to be collected before attending the study. For the baseline measurement (T1), friendship dyads are invited to come to the T1 data collection at their school or to the laboratory at the University of Zurich. All dyads are informed about the study design. In detail they get information that their participation in the study is voluntary. All participants are treated in accordance with the ethical standards of the Declaration of Helsinki [44] that implies that the confidentiality and privacy of all participants is assured all the time (Ethics approval by the Institutional Ethics Review Board of the University of Zurich, Switzerland. Committees reference number: 2017.10.3). Participants are informed that collected data are safely stored and treated as anonymous and that personal codes are used to secure anonymity. Following this information, participants get instructions how to fill in the questionnaires regarding physical activity.

Afterwards, participants get information how to fill in the end-of-day diaries, which is sent to them via e-mail every day for the following 8 days to measure baseline daily diary data. In particular, the friendship dyads are instructed to complete the online questionnaire independently from each other; the daily diaries are to be filled in every night, 1 h before going to bed. To remind about daily diaries participants are prompted every day by automated text messages. In case of non-completion of the diaries for two consecutive days, participants are contacted additionally (e.g. via phone/ email).

After the initial instructions about the study and daily diaries, adolescents complete an online questionnaire with study relevant variables and possible confounders (see measures). In a next step, body weight, height, and body fat is measured with body weight scales and measuring rods. Finally, both adolescents of the friendship dyad are provided with an accelerometer with instructions about how to wear it and charge the battery. The accelerometer should be worn for the following 8 days. The participants do not have to wear it during sleeping hours, bathing, other water activities, or contact sports like martial arts (such missing data regarding PA can be compensated via self-reports of the daily diaries). If questions arise regarding the accelerometers or the daily diary, participants may write an email to the study staff or contact them by phone. After the 8 days of accelerometry, participants are invited for the planning intervention (T2). In addition, all dyads are instructed to not communicate with others about the intervention to avoid interference between the different conditions of the intervention.

#### Intervention procedures (T2)

During the second appointment all dyads are asked about their experience wearing the accelerometer to solve possible problems. In addition, participants return the accelerometer from the first week of wearing. Afterwards, they fill in an online questionnaire with study relevant variables and possible confounders (see measures). At the end of the questionnaire, all participants watch a movie of the Federal Office of Public Health Switzerland of 2:30 min about the benefits of PA, the negative consequences of sedentary behavior, and the recommendation of at least 1 h of moderate to vigorous PA per day (see: https://www.youtube.com/watch?v=ll9m0sx9uAI; this refers to the behavior change techniques (BCT) 5.1 and 5.6 [45]). Thereafter, all participants have to answer a quiz to evaluate whether the given information about PA is recognized by the participants.

After the quiz, the experimenters deliver further education and a motivation treatment. Based on a brochure that all adolescents can take home with them, the experimenter repeats and highlight the guidelines for physical activity. In addition, the experimenter asks for barriers and benefits of physical activity the adolescents perceive (BCT 9.2). Furthermore, the experimenter mention that it is possible to set graded tasks instead of doing 1 h of PA per day in one go (BCT 8.7). Next, the experimenter explains that weight gain might be possible due to physical activity in the beginning of starting being physical active. Furthermore, the experimenter presents how the data of the accelerometer should look like according to the recommendations [4]. As a last part, self-efficacy as an important aspect of goal success is mentioned (BCT 15.1). The materials do not contain any planning statements.

Next the intervention follows (see Fig. 1). Friendship dyads are cluster randomized (see above for further details) to one of the four groups: collaborative experimental planning group, individual experimental planning group, collaborative control group, or individual control group. All dyads are blinded regarding the allocation. In the planning intervention groups, adolescents are instructed to complete a planning sheet with information on how to plan to do more MVPA in their daily lives (see Additional file 1 for an example) whereas the control groups work on a distraction task. All conditions are assisted by the experimenter [7].

##### Individual planning condition

In this condition the adolescents complete the planning sheets on their own. Each adolescent has to develop up to three action plans (BCT 1.4) including when, where and how to be physically active cf. [7]. In addition, each participant should try to anticipate possible barriers for engaging in the planned behavior and plan what he or she could do to overcome these possible barriers (i.e., coping planning [26]; BCT 1.2). Both adolescents are placed at two separate tables to omit any cooperation during the planning task. In addition, they are not allowed to speak to each other. The experimenter is in the room during the planning task.

##### Collaborative planning condition

Adolescents in this condition have to plan together with their friend by discussing when, where and how both members of the dyad are physically active together. In addition, they also have to anticipate possible barriers for engaging in the planned behavior and plan what they could do together to overcome these barriers (i.e., coping planning [26]). In line with the individual planning condition they should write down up to three action and up to three coping plans cf. [7].

##### Control groups

Participants of the two control groups have to interpret a short video showing scenes of different superhero movies (Spiderman and Wonder Woman). Several questions are asked about the characteristics of the two super heroes in the movie and whether these heroes are comparable. Questions have to be answered on a sheet similar to the planning sheets to also keep the time of the distraction task similar to the planning task. In the individual control condition, each participant watches the movie alone and answers all questions by him/herself. Both members of the dyad are not allowed to cooperate and speak to each other. In contrast, participants in the collaborative control condition have to cooperate on the same distraction task. Both members of the dyad watch the movie together and answer the questions conjointly. To control for effects of mere collaboration between the friends, the interaction is structured in a manner analogous to that of the collaborative planning intervention.

After the planning intervention or distraction task, adolescents are provided with an accelerometer which should be worn from the day after the intervention for the following 7 days. After the 7 days, participants return the accelerometers via mail to the University of Zurich.

#### Booster session (T3)

One month after the intervention, the friendship dyads are invited to complete the first follow-up questionnaire at schools. Again, the body weight, height, and body fat is measured. In addition, a booster session cf. [46] of the planning intervention is provided to the participants in the experimental groups. Booster sessions are a repetition of the planning intervention of T2 and augment the long-term impact of planning over a 6-month period cf. [19]. In addition, the friendship dyads have the opportunity to adjust their plans (e.g., due to barriers not yet included in the coping plans [14]) to increase the sustainability of intervention effects. Participants of the two control groups have to rethink their definitions of a hero. Questions have to be answered on a sheet similar to the booster planning sheets.

Afterwards, the body weight, height, and body fat is measured. Next, participants get instructions on how to wear the accelerometer which should be worn from the next day on for the following 7 days. Consequently, the friendship dyads are informed to complete the online questionnaire every evening.

##### Mini-boosters

Both experimental groups receive an e-mail to boost their action plans. These mini-boosters are delivered one, two, and three weeks after the intervention.  In addition, participants receive the e-mails after one, two, three and 4 month of the booster session (T3; see Additional file 2 for an example). Participants need to reply to the e-mail whether they changed their action and coping plans (referring to the plan defined at T2 or in case they revised the plan at the booster session referring to the adapted plan at T3). If yes, they should send the new plan(s) via e-mail to the experimenter. Participants of the collaborative planning condition receive a joint e-mail as mini-booster, whereas those participants of the individual planning condition receive the mini-boosters individually. Participants of the control group get an e-mail with the recommendation of at least 1 h of MVPA per day. That information is also part of the e-mail for the intervention groups.

#### Follow-up (T4)

Five months later (i.e., 6 months after baseline), the friendship dyads are asked to return for a fourth time (T4) for the second follow-up. An online questionnaire with study relevant variables and possible confounders (see measures) is requested to be answered. Furthermore, the body weight, height and body fat is measured. Again participants are instructed to wear the accelerometer which should be worn from the next day on for the following 7 days. In addition, the friendship dyads are asked to complete an online questionnaire every evening. After the end of the whole study, all participants get a debriefing about the aim of the study and the four conditions.

### Incentive

In addition to the reminders via text messages (SMS) during all daily diary phases, participants are offered incentives in order to enhance adherence to the study. First, the friendship dyads get feedback of the Actigraph accelerometer results as well as 50 CHF per person as incentive at the booster session. After completion of the whole study they get an additional 75 CHF per person as a compensation for their time and effort. In addition, all dyads are entered into a prize draw for a chance to win one of at least 8 prizes worth around 2000 CHF in total (e.g., voucher for a theme park, a restaurant, or a climbing tree park). Participants of the pilot study indicated the high attractiveness of this additional incentive.

### Measurements

The primary outcome is the change of MVPA measured with accelerometers. Within this study an ActiGraph wGT3X-BT is used that measures acceleration on three axes, providing a composite measure (i.e., “vector magnitude”). Every participant should wear the personalized accelerometer consistently at the right hip on the mid axillary line [47–49]. In addition, body weight and height, as well as body fat, is measured with standardized scales, i.e. the OMRON BF 214 diagnostic scale to assess secondary outcomes. Further secondary outcomes are assessed via self-reports from both friends of the dyads and are described in Table 1. The main constructs as well as control variables are measured with validated questionnaires. The daily diaries include only single-item measures to keep participant burden low. All questionnaires are implemented via the online survey tool Unipark.

**Table 1:** Main measurements (self-reports)

Construct [References for measurement]	Description	Included in:				
		T1	T2	T3	T4	Diary
The outcome variable, additional assessment						
Physical activity [42, 65]	Duration in minutes (together with the friend), intensity	X	X	X	X	X
Mediator and control variables						
Risk perception [53, 64, 66, 67]	Perceived vulnerability for one’s own health	X		X	X	X
Outcome expectancies [53, 64, 66, 67]	Expected positive and negative consequences of both acting and not acting	X		X	X	X
Self-efficacy (individual and collaborative) [53, 64, 66, 67]	Feeling of competency regarding a person’s ability to overcome barriers regarding PA	X		X	X	X
Intention (Individual and collaborative) [53, 64, 66]	Intended action of PA	X		X	X	X
Action and coping planning (individual and collaborative) [53, 64, 66]	Action planning pertains to the when, where, and how of intended action; coping planning is the anticipation of barriers and the design of alternative actions	X		X	X	X
Action control [53, 64]	Action control includes three subfacets: awareness of intentions, self-monitoring and regulatory effort	X		X	X	X
Received social support from the friend [53, 64, 67]	Emotional and practical received social support regarding PA		X	X	X	X
Provided social support by the friend [53, 64, 67]	Emotional and practical provided social support regarding PA		X	X	X	X
Mobilization of peer social support [53, 64, 67]	Activation of social support from the friend		X	X	X	X
Characteristics of received peer social support [53, 64, 67]	Perceived quality, responsiveness, and satisfaction with social support		X	X	X	X
Received peer social control [68]	Received social control from the friend regarding PA		X	X	X	X

The main control variables include the socioeconomic status (e.g., age, family characteristics like parents’ education), friendship quality and stability, time per week spent with the friend (face-to-face and online/on phone), barriers to perform MVPA, physical activity motives, positive and negative affect, and social desirability. Control variables that are unlikely to vary over time (e.g. socioeconomic status) are measured once at the baseline.

### Data collection and data quality

A team of experimenters (bachelor, master, or PhD students in Psychology or equivalent) conduct data collection. All experimenters are trained by the third author of the study on all aspects of data collection, including consent, and questioning techniques. Furthermore, they are supervised by the third author who monitors data collection quality, and provides on-site feedback. In addition, the third author coordinates the overall project.

### Duration of the project

**Fig. 2:**
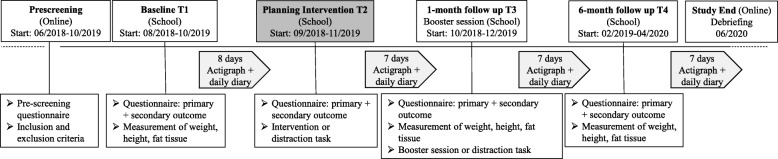
Project duration and timeline of the study

Data collection for the baseline assessment starts in August 2018. Details of the implementation timeline are illustrated in Fig. 2.

### Power analyses

The sample size was calculated by using the G*Power program [50] to secure adequate power. The needed sample size derived from the assumption of a medium effect (*d* = 0.50) of planning on PA as derived from previous research [18, 19, 21]. To detect a significant difference between planning conditions and control conditions at *p* < .05 with a power of 1-β = .80, 256 friendship-dyads are required, equally assigned to the four groups (*n* = 64 per group). Moreover, drop-outs have to be taken into account. Daily-diary studies with children and adolescents report dropout rates between 15 and 56% e.g., [19, 23]. Due to positive feedback from participants of the pilot study and increased efforts to maintain commitment to the study in participants (e.g., sending birthday and seasonal cards and via financial incentives after the third completed point of measurement) we assume a drop-out rate of 20%. Therefore, 308 dyads are required, i.e. 77 dyads per group. For the comparison between the individual planning and the collaborative planning group effect sizes of medium size were reported (e.g., *d* = 0.44, *d* = 0.49 after one and 6 months respectively [30]). To detect a significant difference between the two different planning conditions at *p* < .05 with a power of 1-β = .80, with a given effect size of 0.44, a group size of 83 per group is required, resulting in a total of 332 dyads. Including a dropout rate of 20%, 400 dyads, i.e. 100 per group, will be necessary to ensure adequate power.

Monte Carlo simulation, the recommended method to estimate the sample size for the analyses of intraindividual associations during the diary phase [51] could not be conducted. This is due to the fact that detailed information on parameters from previous studies are required which are not available as this is the first study of its kind. However, this sample size fits to the requirement of Kenny, Kashy, and Cook [52] to test for non-independence of dyadic data structure with continuous outcomes. The minimum required number of dyads allowing testing for consequential nonindependence (e.g., similarity of both adolescents regarding study relevant variables) is 28 friendship dyads per group.

### Data management

All data obtained from the online survey tool Unipark are automatically stored at the online server “Questback”. Afterwards, these data are saved anonymously at a secured server of the University of Zurich. The same procedure applies for all mails of the minibooster sessions. A personal code is used to secure anonymity. All contact details including names and telephone number are stored separately from the data set. Data of the personalized ActiGraph devices are coded with the same personal codes and stored in the same secured folder as the online questionnaires. Only authorized persons like author 1 and 3 have access to the data set. All data obtained during the planning sessions (i.e. planning sheets) as well as the data concerning weight, height, and body fat are stored in a locked storage. One experimenter enters the data into an SPSS data file. All entries are double checked by a second experimenter to correct possible errors during data entry.

### Data analyses

Data are analyzed by the first and third author after consultations with other authors. Accelerometer-based PA data is analyzed with the program Actilife 6.13.3 (ActiGraph, Pensacola, FL). Data are screened separately for each participant in order to identify spurious data or monitor malfunctioning. For the present study, only days with at least 10 h of wear time are considered as valid and included into analyses [53]. The accelerometer physical activity data are assessed at a frequency of 30 Hz and reintegrated into 60 s-epochs for data processing. Non-wear time is filtered using an automated algorithm [54]. For the analysis, the output of acceleration counts on three axes, MVPA, energy expenditure, metabolic equivalent of task (MET), and steps are evaluated. For the cut points of acceleration and MVPA, the settings by Evenson et al. are used [55]. For each participant, the total minutes in MVPA per day is calculated based on the threshold by Evenson et al. [55]. The energy expenditure is calculated according to the recommendation of Freedson and colleagues [56] and the MET value is assessed with the Algorithm by Freedson et al. [57] as it was developed for younger people.

Analyses of the hypothesized differences between the groups (see the trial registration: ClinicalTrials.gov: NCT03575559.) use the mean scores of accelerometer measured moderate-to-vigorous physical activity (frequency and duration) across the seven-day periods and for the dyads and compare the planning groups with the control groups. Mediator analyses for the intervention effects are done by means of regression analyses [58]. To analyze intervention effects at the between- and within-person level on a daily basis multilevel modelling is used [59]. Data analyses of the daily diary data in indistinguishable dyads are conducted using structural equation modelling with equality constraints as suggested by Olsen & Kenny [60].

### Dissemination plan

All findings are presented in international scientific conferences, and submitted for peer-reviewed international publication.

## Discussion

The study offers an essential step for understanding the effectiveness of two planning interventions and their underlying mechanisms in adolescent friendship dyads. By investigating a planning intervention of PA with daily diaries, long-term follow-ups and devise-based measurement of MVPA, the study significantly contributes to our knowledge on planning interventions in adolescents. In addition, this project makes an important contribution to a previously neglected research area by comparing collaborative planning with individual planning in adolescents. In doing so, this project is of great theoretical and practical significance. From a theoretical perspective, the findings provide an insight into the effectiveness of individual and collaborative planning and their underlying mechanisms in everyday life as well as during longer-term follow-ups while applying state of the art measurements of PA. Moreover, investigating potential differences in the effects of individual and collaborative planning substantially further our knowledge on the most effective planning intervention for adolescents. Thus, it is possible to draw conclusions for practical interventions for adolescents who are in need of preventive or treatment actions, improving their PA and reducing sedentary behaviors. Recommendations can be given as to whether or not planning facilitates the engagement of MVPA in adolescents and which planning intervention might be the most promising one to follow.

Nevertheless, there are some additional critical points regarding the study protocol. One is that the individual and collaborative planning interventions comprises several components, i.e. the education and general motivational treatment in addition to the planning intervention. However, this is in line with other planning interventions e.g., [61] to secure that differences in knowledge or motivation do not interfere with the intervention itself. Another potential limiting factor regarding the investigation of the effect of the planning intervention might be the possible confounding effect of the assessment of the self-reported PA on a daily basis. The instruction to all individuals to report all incidents of moderate-to-vigorous physical activity via daily diaries is likely to also trigger self-monitoring, which is known to be an effective strategy to change behaviour [62]. Furthermore, all individuals get feedback about the Actigraph results at the booster session and the last time point of measurement. Even though all participants just get a graphical representation of the results without any statement, whether they reached the recommended amount of PA of 1 h per day (BCT 2.2), this might affect their behavior. In line with this, the measurement of body weight and fat is also not blind which means that participants get a feedback about their weight (BCT 2.2). It also has to be mentioned that the use of accelerometers, although blinded to participants, might contribute to mere measurement effects. It is possible that the wearing of accelerometers prompts participants to increase physical activity already at baseline even though they are instructed to behave as usual cf. [61].

Another important point regarding the present study concerns the successful recruitment of friendship dyads meeting all the inclusion criteria. The recruitment and the data collection of 400 dyads is demanding, but realizable compared with other studies which investigated dyads with more restrictive inclusion criteria [63]. Moreover, in our pilot study, 20 dyads were recruited in less than 1 month (January 2016). The pilot study included a 7 days diary phase and a follow-up. Although the full design for the proposed project is much more demanding, experience from our pilot study indicates that recruitment of large numbers of friendship dyads via schools is feasible. However, the difficulties with the recruitment of dyads needs to be kept in mind when evaluating the sample size of studies including dyads. In line with Scholz & Berli [64] we therefore assume a position where the primary focus is on effect sizes, not on significance testing. Despite these challenges outlined above, this study has a potential to substantially further our knowledge with regard to planning interventions in adolescents.

## Additional files

Additional file 1: Example of the planning sheet. (DOCX 59 kb).

Additional file 2: Example of the mini booster. (DOCX 49 kb).

## Abbreviations

CP: Collaborative planning
IP: Individual planning
MVPA: Moderate-to-vigorous PA
PA: Physical activity
RCT: Randomized control trial

## Footnotes

1 In the current paper both terms are used interchangeable as done by many researchers. However, slight differences, based on their origin, exist in the definition of both strategies. For a detailed description of the differences see [7]. Overall, it is proposed that action planning is a good planning intervention to reach a broader and more complex set of behavioral responses, e.g., like an increase in PA.

2 The term conjoint planning is used to comprise collaborative and dyadic planning.
