# Supplementary material for: A cluster randomized controlled trial comparing the effectiveness of an individual planning intervention with collaborative planning in adolescent friendship dyads to enhance physical activity (TWOgether)
Source: BMC Public Health. 2018 Jul 24;18:911. doi: 10.1186/s12889-018-5818-6 (PMC6056914; doi:10.1186/s12889-018-5818-6)
Supplement: Supplementary file 1 — Example of the planning sheet. (DOCX 59 kb). [file 12889_2018_5818_MOESM1_ESM.docx]

**Additional file 1**

**Example of the planning sheet.**

**Instructions and examples**

Please read these instructions.

Many people decide to be more active in sport, but often forget this intention. It has been found that plans help to act according to the intention. In addition, you will be more active if you formulate very concrete plans, WHEN, WHERE and HOW you want to be physical active.

It is therefore helpful for you to plan on which weekdays, at what time and on which occasions you want to be active in sports over the next 7 days. The more precise, concrete and personal you formulate these plans, the more they will help you!

Example:

Plans could look like this:

"If it's Saturday 11:00 o`clock, then I'll cycle around the lake “Pfäffikersee” for 20 minutes."

Or

"If school is out (every day from Mo to Fri), then I go jogging in the forest for 30 minutes."

For these examples, the following would be entered in the table:

| WHEN  (Time) | | WHEN (Weekday(s)) | WHERE  (places/occasions) | HOW  (type of sport) | HOW long (in minutes) |
| --- | --- | --- | --- | --- | --- |
| During the next 7 days, I will be physically active at the following times... | | ...on the following weekday(s)... | .... at the following places / opportunities... | ...in this way and for... | … ___minutes. |
| 1. | at **11:00** o’clock | **Saturday** | **Pfäffikersee** | **Ride my bycicle** | **20 minutes** |
| My 1st plan:  If it is Saturday 11.00 o'clock, then I cycle around the lake Pfäffikersee for 20 minutes. | | | | | |
| 2. | **After school** | **Mo to Fr** | **forest** | **Jogging** | **30 minutes** |
| My 2nd plan:  If school is out, then I go jogging in the forest for 30 minutes. | | | | | |

**Now it’s your turn**

Now think about **WHEN** (weekday/time) you want to be physically active during your free time that you get out of breath and start sweating. Afterwards, **WHERE** (place/what opportunity) you want to be physically active and **HOW** (sport/duration) you want to be physically active. Please consider up to **3 plans** and make sure that you are physically active (including school sports, sports in a club, etc.) for at least 60 minutes per day. You can collect your ideas on a piece of paper.

Please note that a good plan for physical activity should meet 4 criteria:

1. Do your plans fit to you?
2. Do your plans help you to be physically active for at least 60 minutes per day?
3. Can your plans be integrated into your daily routine?
4. Are your plans accurate and complete?

Please formulate your plans in a way that you can answer all questions with Yes.

| WHEN  (Time) | | WHEN (Weekday(s)) | WHERE  (places/occasions) | HOW  (type of sport) | HOW long (in minutes) |
| --- | --- | --- | --- | --- | --- |
| During the next 7 days, I will be physically active at the following times... | | ...on the following weekday(s)... | .... at the following places / opportunities... | ...in this way and for... | … ___minutes. |
| 1. | At ________ o’clock |  |  |  |  |
| My 1st plan:  If________________________, then_____________________________________ | | | | | |
|  | | | | | |
| 2. | At________ o’clock |  |  |  |  |
| My 2nd plan:  If________________________, then_____________________________________ | | | | | |
|  | | | | | |
| 3. | At________ o’clock |  |  |  |  |
| My 3rd plan:  If________________________, then_____________________________________ | | | | | |

If you have generated your plans, please write down **When**, **Where** and **How** you want to be physically active in the boxes below. Afterwards, please formulate your plans according to the If-Then format (If situation X is..., then I will initiate Y.). Please also see the examples on page 1. If you need any help or if you have no ideas, please contact the experimenter.

*As soon as you finished your plans, please contact the experimenter.*

**Instructions and examples**

It is particularly useful, in addition to your plans, if you consider which situations could make it difficult for you to transfer your planned physical activities into action and how you can handle such critical situations.

Please write down which situations could most compromise your plans described above (e.g. overlapping appointments or broken sports equipment) and how you could handle such situations to still translate your plans into action.

Example:

*"****If*** *my bike is broken,* ***then*** *I'll go swimming in the indoor pool in town for 30 minutes."*

*"****If****, for once, I don't have time after school,* ***then*** *I go skating for 30 minutes in the evening after finishing my homework."*

For these examples, the following would be entered in the table:

| Situation | | HOW? | |
| --- | --- | --- | --- |
| What barriers can occur? | | How can you handle this situation? | |
| 1. | **Broken bike** | 1. | **Swimming in the indoor pool** |
| My 1st plan:  If my bike is broken, then I go swimming for 30 minutes in the indoor pool in town. | | | |
| 2. | **No time after school** | 2. | **Alternative date after homework** |
| My 2nd plan:  If I don't have time after school, then I go skating for 30 minutes in the evening after finishing my homework. | | | |

**Now it’s your turn**

First think about critical situations or barriers that could make it difficult for you to translate your planned sports activities into action.

Afterwards consider how you could handle these critical situations/barriers and write down your plans. Again, you can first collect ideas and write them down on a piece of paper.

**The more precise, concrete and personal you formulate these plans, the more they will help you!**

Your plans should meet three criteria.

1. Are these really your main barriers for your plans?
2. Are your solutions suitable to overcome the barriers?
3. Can your plans be integrated into your daily routine?

Please formulate the plans so that you can answer all questions with Yes.

If you have generated plans, please write down what **barriers** might occur and how you can **handle these situations** in the boxes below. Afterwards, please formulate your plans according to the **If-Then format** (If situation X is..., then I will initiate Y.). Please also see the examples on page 3. If you need any help or if you have no ideas, please contact the experimenter.

| Critical situations | | HOW? | |
| --- | --- | --- | --- |
| Which barriers can occur? | | How can you handle this situation? | |
| 1. |  | 1. |  |
| My 1st plan:  If______________________________________________,then_________________________________. | | | |
|  | | | |
| 2. |  | 2. |  |
| My 2nd plan:  If______________________________________________,then_________________________________. | | | |
|  | | | |
| 3. |  | 3. |  |
| My 3rd plan:  If______________________________________________,then_________________________________. | | | |

*As soon as you finished your plans, please contact the experimenter.*
