# Supplementary material for: A cluster randomized controlled trial comparing the effectiveness of an individual planning intervention with collaborative planning in adolescent friendship dyads to enhance physical activity (TWOgether)
Source: BMC Public Health. 2018 Jul 24;18:911. doi: 10.1186/s12889-018-5818-6 (PMC6056914; doi:10.1186/s12889-018-5818-6)
Supplement: Supplementary file 2 — Example of the mini booster. (DOCX 49 kb). [file 12889_2018_5818_MOESM2_ESM.docx]

**Additional file 2**

Example of the mini booster.

Dear [Name],

We would like to remind you to be physically active for at least one hour per day.

We also ask you to consider whether your plans still fit to you. Are they still realistic, can they be integrated into your everyday life and help you to be physically active? Or have barriers occurred that you have not considered before?

Please consider whether you want to adjust your plans.

YES: If you want to adapt them, just fill in the empty handout "My Plans" you received from us. Please proceed in the same way as you did at our last appointment. Note the following questions:

1. Do your plans fit to you?
2. Can your plans be integrated into your daily routine?

Please send us a photo or even better a scan of your new plan(s) by e-mail. Please enter your name and the name of your school.

NO: If your plans are still suitable for you, please write us a short mail that you do not adapt the plans.

Thank you very much!

Your TWOgether team
